# Low-Energy Plasma Focus Device as an Electron Beam Source

**DOI:** 10.1155/2014/240729

**Published:** 2014-07-21

**Authors:** Muhammad Zubair Khan, Yap Seong Ling, Ibrar Yaqoob, Nitturi Naresh Kumar, Lim Lian Kuang, Wong Chiow San

**Affiliations:** ^1^Plasma Technology Research Center, Department of Physics, Faculty of Science, University of Malaya, 50603 Kuala Lumpur, Malaysia; ^2^Department of Physics, Federal Urdu University of Arts, Science & Technology, 45320 Islamabad, Pakistan; ^3^Faculty of Computer Science and Information Technology, University of Malaya, 50603 Kuala Lumpur, Malaysia

## Abstract

A low-energy plasma focus device was used as an electron beam source. A technique was developed to simultaneously measure the electron beam intensity and energy. The system was operated in Argon filling at an optimum pressure of 1.7 mbar. A Faraday cup was used together with an array of filtered PIN diodes. The beam-target X-rays were registered through X-ray spectrometry. Copper and lead line radiations were registered upon usage as targets. The maximum electron beam charge and density were estimated to be 0.31 *μ*C and 13.5 × 10^16^/m^3^, respectively. The average energy of the electron beam was 500 keV. The high flux of the electron beam can be potentially applicable in material sciences.

## 1. Introduction

Dense plasma focus (DPF) is potential candidate for various technological applications. The emission characteristics of electron beams and X-rays, as well as the correlation of the electron and X-ray pulses with other PF phenomena, have been previously reported [1–5]. Pulsed electron beams and X-rays should be analyzed to explore possible application-oriented studies and elucidate the physical phenomena responsible for the generation and acceleration of charged particles and the emission of electromagnetic radiation pulses. Several studies have focused on the physical mechanisms of the generation of electron beams by correlations [6] with HXR emission [7], ion beam [8], neutron [9], and electrical measurements [10].

Tartari et al. [11] proposed X-ray brachytherapy sources based on the interactions of the relativistic electron beam of DPF with a high-Z target. The electron beam energy was distributed from nearly 20 keV to 500 keV using a magnetic spectrometer [12]. Tartari et al. [13] measured the X-ray spectra in a 7 kJ DPF device and hypothesized that the electron beam responsible for X-ray production comprised relativistic electron beams and electrode components with energies of about 30–45 keV. Time-resolved studies of electron beam emission were carried out using different detectors, such as Cherenkov detector [14], Rogowski coil [15], Faraday cup [16–18], and magnetic energy analyzer [19]. Faraday cup is a cost-effective and simple detector that exhibits fast signal processing and particle detection with an energy range of a few keV to hundreds of keV.

This study aimed to explore the use of low-energy plasma focus devices as sources of electron beam. These devices were characterized by high electron beam energy and high flux, which could be beneficial for applications in material sciences. Analysis of the X-ray spectra with lead target revealed the marked effects of electron beams in our plasma focus device.

## 2. Experimental Setup

**Figure 1:**
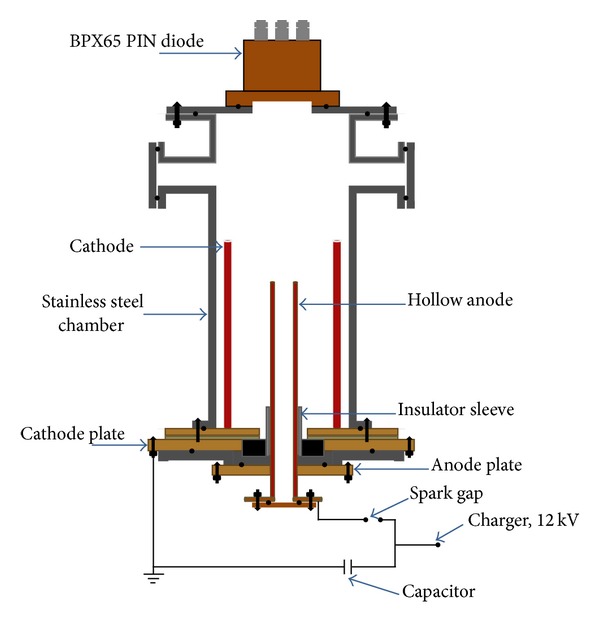
Schematic of plasma focus device.

Experimental measurements were carried out on a Mather-type plasma focus device. This device was energized by a 30 *μ*F Maxwell capacitor, which was charged up to 12 kV with a lead target. Lead material with diameter and width of 1.5 cm and 0.35 cm, respectively, was used. The calculated total external inductance was found to be 165 nH.  Figure 1 shows the diagram of a 2.2 kJ plasma focus device.

The discharge tube comprised copper electrodes, in which the inner electrode exhibited a hollow cylindrical form and served as an anode with diameter and effective length of 1.9 cm and 18 cm, respectively. Hollow anodes were used considering previous studies on energetic electron beams in plasma focus devices. The outer electrode consisted of six copper rods in the form of a squirrel cage with an inner diameter of 3.2 cm. The length of the individual cathode rod was 27 cm, which was 9 cm higher than that of the anode rod. Pyrex glasses were used as insulators to separate the hollow anode and cathode. The vacuum system comprised a rotary vane pump and an evacuated chamber with a pressure lower than 10^−2^ mbar, which was sufficient for experimentations. The chamber was refreshed after every shot to reduce gas contamination with impurities on the output radiation. Fresh Argon gas was refilled to a desired pressure.

Identical coaxial cables (length, 110 cm) were used for all electrical diagnostics. All coaxial cables were protected with aluminum foils to reduce the effects of electromagnetic noises on data signals. Two DPO4043 digital storage oscilloscopes were used to record all electrical signals from the Rogowski coil, high-voltage probe, five-channel PIN diode, and energetic electron beams through the Faraday cup. The oscilloscope was simultaneously triggered for all electrical signals.

Five-channel PIN diodes were normalized against each other by masking with identical foils of aluminized Mylar (thickness, 23 *μ*m). The PIN diode response ranged between 1 keV and 30 keV. Filter combinations were used to verify the variations in the spectral window.

**Figure 2:**
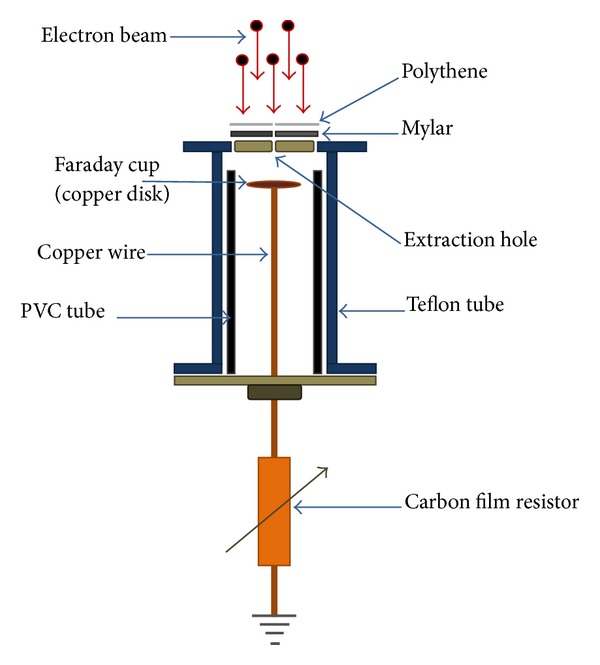
Schematic of Faraday cup.

The Faraday cup contained a metallic disk that served as a charge collector and developed in bias mode with a resistance of 0.1 Ω, which was prepared with a parallel combination of 10 carbon film resistors (resistance, 1 Ω). The Faraday cup comprised a flat circular copper disk (diameter, 7.5 mm) placed inside a Teflon insulator tube, which was then enclosed in the PVC pipe (Figure 2).

A small extraction hole (diameter, 3 mm) was placed in front of the disk covered with Mylar and polythene sheet to allow only the axial movement of energetic electrons that hit the copper disk and reduce the effects of high potential and radiation on the signals of the energetic electron beam before and after plasma focus. The diameter of the Faraday cup was restricted by the diameter of the PVC insulator tube, which was used inside the hollow anode. The energetic electron beam was detected by placing the Faraday cup at the bottom end of the hollow anode at a distance of 37 cm from the anode tip.

**Figure 3:**
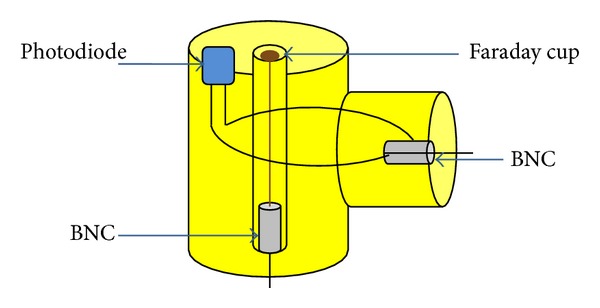
Schematic of arrangement of Faraday cup with photodiode.

A photodiode (BPX65) was used with the Faraday cup at the same position and distance from the top end of the hollow anode (Figure 3).

We proposed a technique to identify the emissions from the electron beam with photoemissions before and after focusing from the focus region.

**Figure 4:**
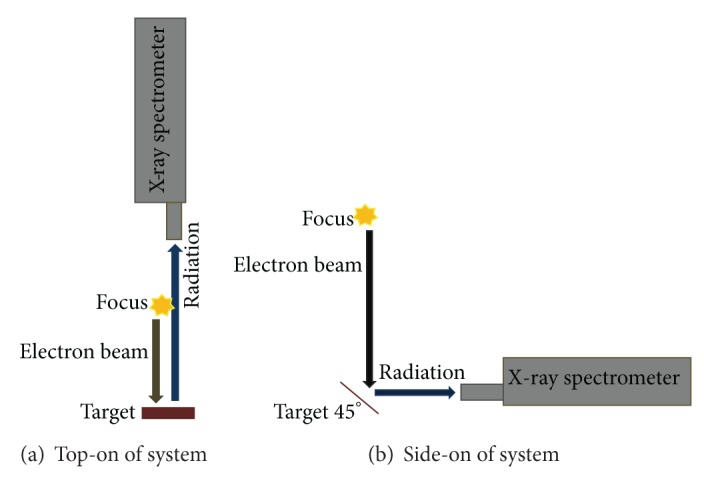
X-ray spectrometer position at top-on of system with target (a) and side-on of system with target at an angle of 45 degrees (b).

An XR100CR X-ray spectrometer was used at the top and side of the system to record the X-ray line spectra caused by the electron beam-lead target collision (Figure 4).

This spectrometer was suitable for analyzing X-ray energy distribution and was sensitive up to 45 keV energy.

## 3. Results and Discussion

We analyzed the electron beam emissions from the low-energy plasma focus device to demonstrate that the charged particles are normally emitted from the plasma column. The directions of the emission of ions and electrons are opposite each other and directed toward the anode for the electrons. The emitted electron beams exhibit energies ranging from few keV to hundreds of keV [20, 21].

To assess the use of low-energy plasma focus devices as electron beam sources, various parameters were analyzed with respect to the filling pressure of the Argon gas. X-ray emissions from Argon-operated plasma focus were investigated by time-resolved PIN diode detectors.  Table 1 lists the design parameters of the plasma focus device.

An array of filtered five-channel PIN diodes was housed at a distance of 43.50 cm from the head of the hollow anode to detect X-rays and measure the radiation emission from the plasma focus. The glass window of the PIN diode was covered with Al foils with specific thicknesses (Table 2) and was detached to detect X-ray emissions. The PIN diode response ranged from 0.5 keV to 30 keV [22].

**Figure 5:**
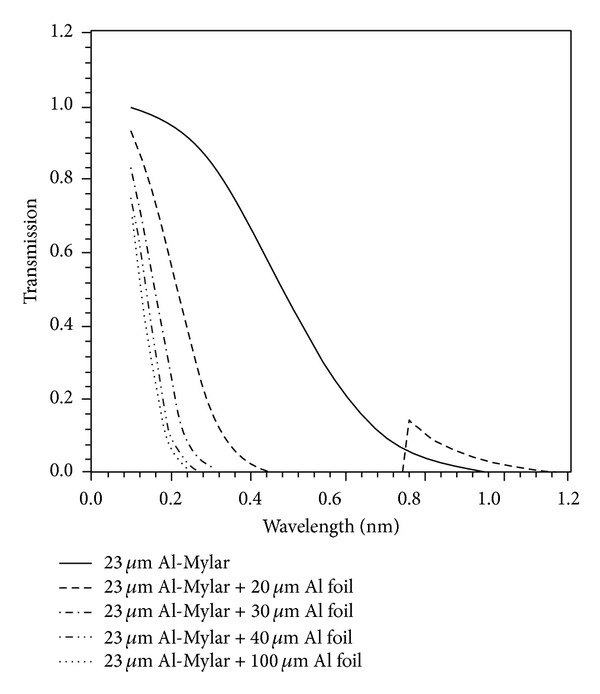
Transmission curves of 23 *μ*m aluminized Mylar, 23 *μ*m aluminized Mylar + (20 *μ*m, 30 *μ*m, 40 *μ*m, and 100 *μ*m) Al foil.

Figure 5 illustrates the transmission curves of the BPX65 diode that was attached with absorption filters.

The X-ray yield in 4*π*-geometry and the system efficiency of X-ray emission could be calculated from five-channel PIN diodes that were masked with Al foils. The X-ray yield was calculated as follows [23–25]:

(1) $$\begin{array}{c} Y=\frac{Q_{\exp}\left(4\pi \right)}{d\Omega S\left(E\right)T\left(E\right)}, \end{array}$$

where

(2) $$\begin{array}{c} Q_{\exp}=\int \frac{V\;dt}{R}\left(\text{C}\right). \end{array}$$

∫*V* *dt* represents the area under the curve with the filter of the PIN diode, *R* = 50 Ω, *S*(*E*) is the average sensitivity of the detector, and *T*(*E*) is the average filter transmission. *d*Ω = *dA*/*r*_*o*_^2^ (sr.) is the solid angle subtended by the detector at the anode center, where *dA* = *πr* ^2^, *r* is the radius of the exposed area of one detector, and *r*_*o*_ is the distance between the detector and hollow anode.

The results were discussed in three parts as follows.

**Figure 6:**
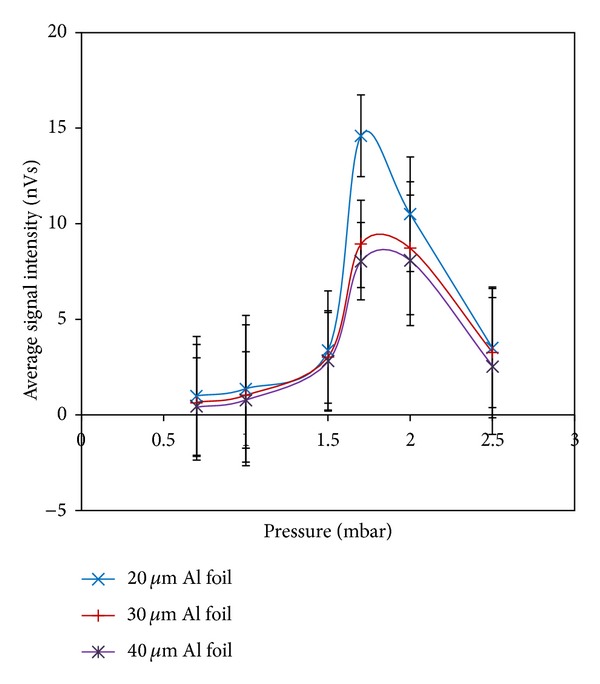
Variation of average signal intensity recorded by Al foil (20 *μ*m, 30 *μ*m, and 40 *μ*m) versus Argon gas pressure.

*(1) Result with Five-Channel PIN Diode*. The variations in X-ray emissions as functions of the Argon gas pressure possess efficient functions in generating radiation in the plasma focus device. In our experiment, a pair of Ross filters (20 *μ*m Al foil, 100 *μ*m Al foil; 30 *μ*m Al foil, 100 *μ*m Al foil; and 40 *μ*m Al foil, 100 *μ*m Al foil) were used to determine the X-ray yield.  Figure 6 shows the variations of the average signal intensity with different Argon gas pressures.

The maximum average signal intensities were recorded with 20 *μ*m, 30 *μ*m, and 40 *μ*m Al foil at an Argon gas pressure of 1.7 mbar.

**Figure 7:**
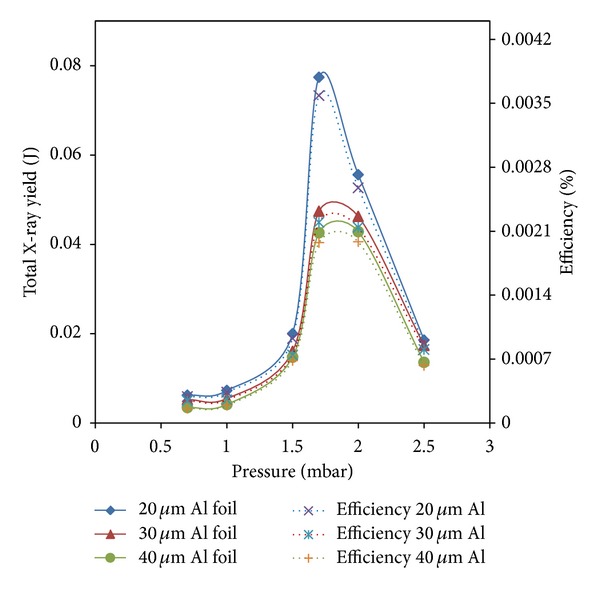
Variation of total X-ray yield in 4*π*-geometry with efficiency versus Argon gas pressure at constant applied voltage 12 kV.

The variations in the total X-ray yield and efficiency against Argon gas pressures at a constant applied voltage of 12 kV are shown in Figure 7.

The maximum total X-ray yields were 77 mJ, 47 mJ, and 42 mJ with corresponding efficiencies of 0.0035%, 0.0021%, and 0.0018%, respectively, in 4*π*-geometry at an optimum pressure of 1.7 mbar. After a series of experiments, we obtained maximum X-ray yield at a pressure of 1.7 mbar at a constant voltage of 12 kV by using pairs of Al foils with specific thicknesses (20 *μ*m Al foil, 100 *μ*m Al foil; 30 *μ*m Al foil, 100 *μ*m Al foil; and 40 *μ*m Al foil, 100 *μ*m Al foil). These foils were fixed on top of the hollow anode tip at a distance of 43.50 cm. The energetic electron beam interacted with the lead target, which was placed at a depth of 27 cm in the hollow anode.

The results revealed that the maximum total X-ray yields were 77 mJ, 47 mJ, and 42 mJ at an Argon gas pressure of 1.7 mbar by using pairs of Al foils with respective thicknesses of 20 *μ*m and 100 *μ*m, 30 *μ*m and 100 *μ*m, and 40 *μ*m and 100 *μ*m. The results from our low-energy plasma focus device were significant. The radiated energy depended on the filling pressure and the shape of the hollow anode because of the strong electron beam-hollow anode interactions. The X-ray yield decreased for pressures higher or lower than the optimum Argon pressure (1.7 mbar). The X-ray yield could be enhanced by reducing the system inductance, system size, and factors related to the electron beam-hollow anode interactions. The X-ray yield was high in the Argon pressure range of 1.5 mbar to 2.0 mbar; therefore, the intensity of the electron beam was high in this range for potential applications in analysis of material characteristics.

**Figure 8:**
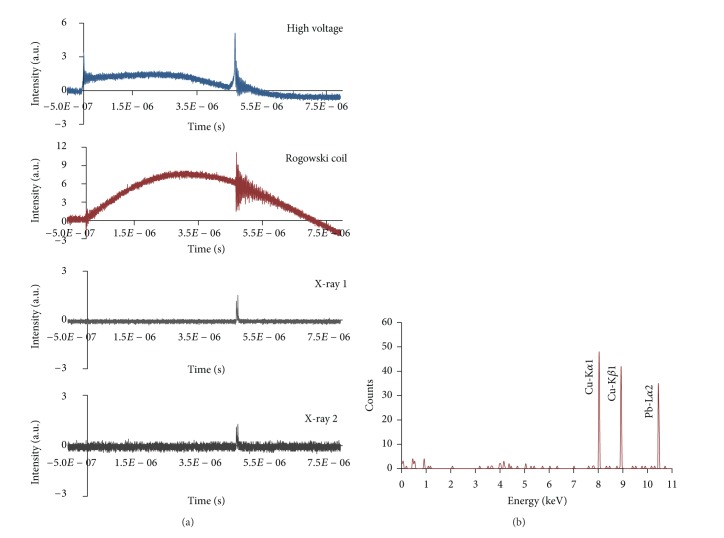
(a) Temporal evolution of X-ray pulses with Al foil thickness for pressure 1.7 mbar, X-ray 1 (23 *μ*m aluminized Mylar plus 30 *μ*m Al foil) and X-ray 2 (23 *μ*m aluminized Mylar plus 20 *μ*m Al foil) with typical high voltage and Rogowski coil signal. (b) X-ray spectrum: X-ray produced by energetic electron beam-target effect at an angle of 45° when the spectrometer is at side-on position.

*(2) Results with X-Ray Spectrometer*. The X-ray spectrometer was used to trace the X-ray line spectra and analyze the X-ray energy spectrum within 45 keV. In the first experiment, this spectrometer was used at side-on position below the system (distance from focus region, 37 cm; distance from the lead target, 4 cm; and angle, 45°). The X-ray line spectra show energies of 8.07 keV, 8.67 keV, and 10.42 keV, which correspond to Cu-K_*α*1_, Cu-K_*β*1_, and Pb-L_*α*2_ lines with temporal evolution of X-ray pulses at specific Al foil thickness and Argon pressure of 1.7 mbar. X-ray 1 (23 *μ*m aluminized Mylar plus 30 *μ*m Al foil) and X-ray 2 (23 *μ*m aluminized Mylar plus 20 *μ*m Al foil) were used with signals from the high-voltage probe and Rogowski coil (Figure 8).

**Figure 9:**
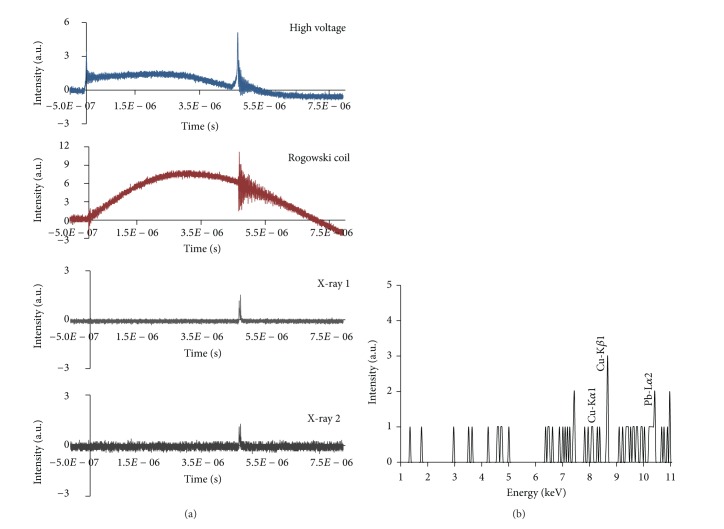
(a) Temporal evolution of X-ray pulses with Al foil thickness for pressure 1.7 mbar, X-ray 1 (23 *μ*m aluminized Mylar plus 30 *μ*m Al foil) and X-ray 2 (23 *μ*m aluminized Mylar plus 20 *μ*m Al foil) with typical high voltage and Rogowski coil signal. (b) X-ray spectrum: X-ray produced by electron beam-target effect when spectrometer is at top-on position.

For the second experiment, the X-ray spectrometer was used at the top-on position of the system at a distance of 47.50 cm from the lead target within the hollow anode. The X-ray line spectra reveal energies of 8.80 keV and 10.46 keV, which correspond to Cu-K_*β*1_ and Pb-L_*α*2_ lines with temporal evolution of X-ray pulses with specific Al foil thickness and Argon pressure of 1.7 mbar. X-ray 1 (23 *μ*m aluminized Mylar plus 30 *μ*m Al foil) and X-ray 2 (23 *μ*m aluminized Mylar plus 20 *μ*m Al foil) were used with signals from the high-voltage probe and Rogowski coil (Figure 9).

Spectrometer results indicated the presence of an energetic electron beam emitted from the focus region because of the instabilities that occur upon hitting the target material. The energy of the energetic electron beam was high enough to produce Pb-K_*α*1_ (74 keV) line radiation in the X-ray line spectra. However, this radiation was impossible to be detected by our spectrometer because of its constraints in the energy range.

**Figure 10:**
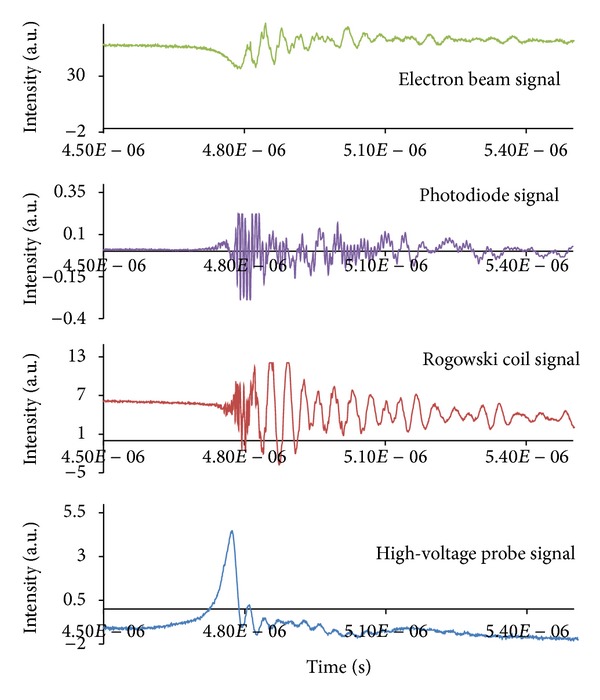
Typical energetic electron beam signal with the signals of photodiode, high-voltage probe, and Rogowski coil (at pressure of 1.7 mbar).

*(3) Results with Self-Biased Faraday Cup*. In this experiment, the Faraday cup and photodiode were employed and uniquely arranged to determine the temporal behavior of the emissions from the electron beam and photoemissions from the plasma focus. Signals from the electron beam, photoemission, high-voltage probe, and Rogowski coil for a shot at a pressure of 1.7 mbar are shown in Figure 10. The electron beam current abruptly increased and coincided with the dip from the signals of the high-voltage probe signal.

For a shot at pressure of 1.7 mbar and electron beam velocity of 4.6 × 10^7^ m/s, the electron beam energy was calculated using the time-of-flight technique from the top part of the hollow anode to the electron collector plate. The calculated average energy of the energetic electron beam was 500 keV at the optimum Argon gas pressure of 1.7 mbar. Therefore, the emissions from the energetic electron beam in the low-energy plasma focus device were significant and were caused by instabilities.

**Figure 11:**
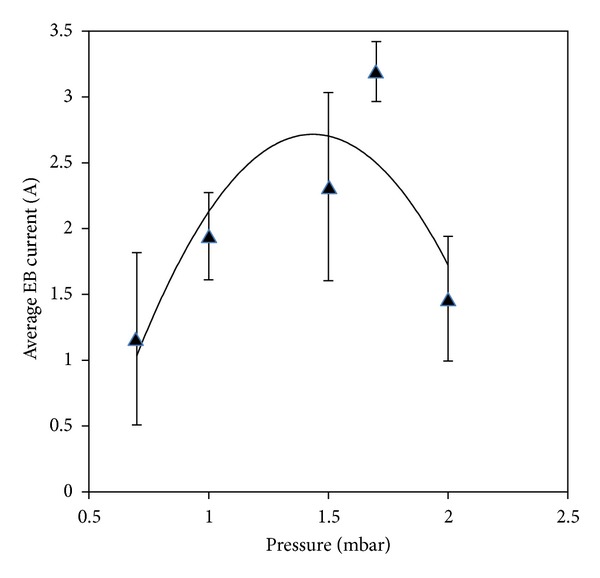
Variation of average electron beam current versus Argon gas pressure.

To study the low-energy plasma focus device as an electron beam source, we explored the parameters that were dependent on the filling pressure of Argon gas. Given the dependence of the average electron beam current and energy with the filling pressure, electron beam signals were recorded from 0.7 mbar to 2.0 mbar.  Figure 11 indicates that the electron beam average current is strongly dependent on the filling pressure.

The maximum average current of the electron beam was 3.19 A obtained at the optimum pressure of 1.7 mbar. At this pressure, the device favors the appropriate discharge dynamics to form a strong pinching [26]. The pinching time occurs near the maximum discharge current, thereby transferring the maximum energy into the plasma. As a result, the emissions from the electron beam were optimal at this pressure, and the maximum average current of the electron beam was achieved. Below the optimum pressure, the beam current decreased because of the weak dynamics of the current sheath at low gas pressures. Increasing the Argon pressure beyond the optimum value decreased the velocity of the current sheath because of the increased sheath mass. Therefore, the focus formation weakened and low electron emissions were produced.

**Figure 12:**
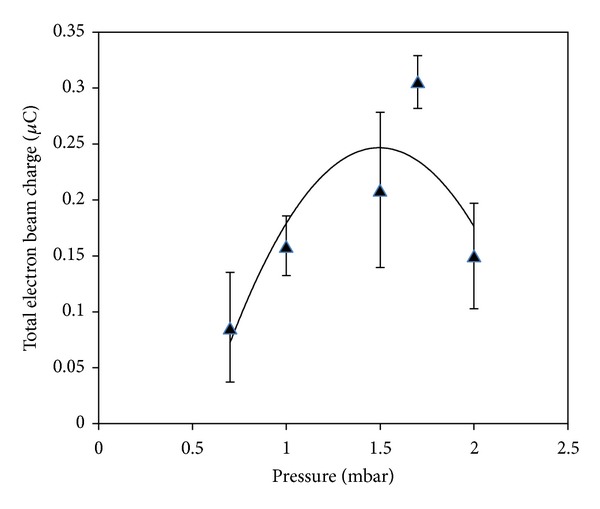
Variation of total electron beam charge versus Argon gas pressure.

**Figure 13:**
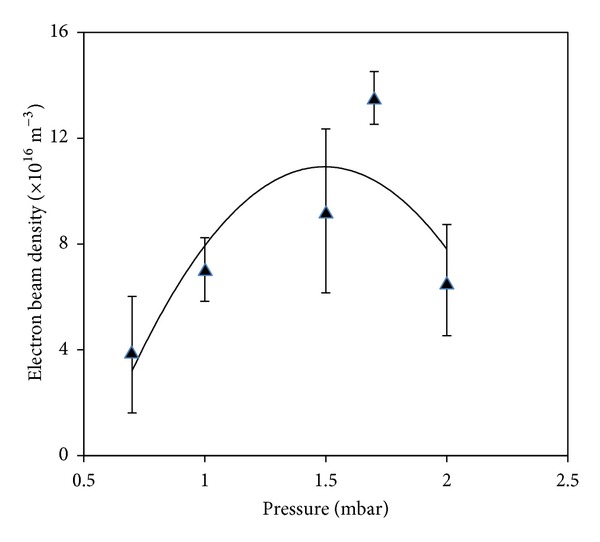
Variation of electron beam density versus Argon gas pressure.

Figures 12 and 13 show the variations in the charge and density of the electron beam with the filling pressure of Argon gas, respectively.

The charge and density of the electron beam were pressure dependent and, respectively, reached their maxima of 0.31 *μ*C and 13.5 × 10^16^/m^3^ at the optimum pressure of 1.7 mbar.

**Figure 14:**
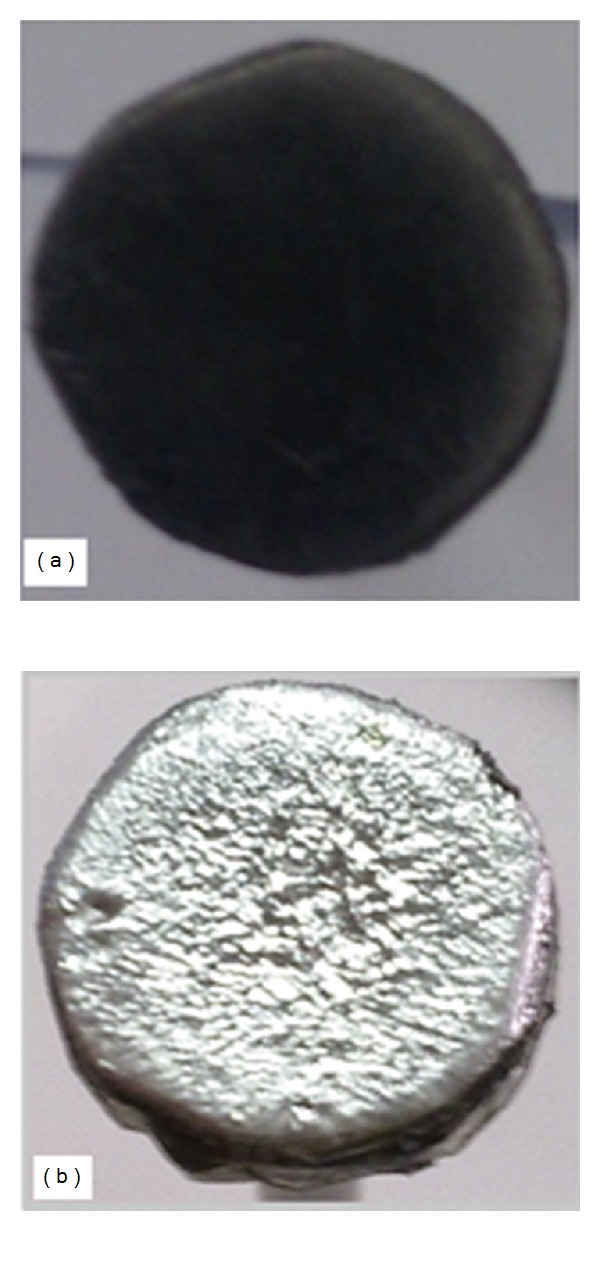
Images of the lead target (a) before and (b) after interaction with electron beam in the plasma focus.

The images of the target before and after being hit by the electron beam in the plasma focus are shown in Figure 14; this interaction is attributed to the bombardment of the energetic electron beam.

The images reveal that when the electron beam hits the lead target (diameter, 1.5 cm; width, 0.35 cm), the electron beam is generated from the plasma focus region. Lead is a feasible material to produce maximum X-ray yield, in contrast to other materials. The marked image in Figure 14(b) is attributed to the interaction of the target with electron beam generated for Argon pressures ranging from 1.5 mbar to 2.0 mbar. The flux of the electron beam significantly affected the material.

The results of X-ray yields and average energetic electron energies were achieved at a pressure range of 1.5–2.0 mbar because of the strong bombardment of the electron beam. Furthermore, the results from the X-ray spectrometer supported the emissions of the energetic electron beam from the focus region because of instabilities upon hitting the target material. The flux of the electron beam significantly affected the material.

The lower and upper limits of the electron energy differed from the actual values because of the technical limitations of our approach. The proposed technique provided appropriate information of low-energy plasma focus devices as electron beam sources.

## 4. Conclusions

A combination of Faraday cup and photodiode was used to investigate a low-energy plasma focus device (2.2 kJ, 12 kV) as an electron beam source at an Argon gas pressure of 1.7 mbar. The average electron beam energy was found to be 500 keV with the self-biased Faraday cup. The maximum charge and density of the electron beam were 0.31 *μ*C and 13.5 × 10^16^/m^3^, respectively. The maximum total X-ray yields were 77 mJ, 47 mJ, and 42 mJ with corresponding efficiencies of 0.0035%, 0.0021%, and 0.0018%, respectively. The X-ray line spectra revealed Cu-K_*α*1_, Cu-K_*β*1_, and Pb-L_*α*2_ lines with the side-on position of the lead target at 45°; Cu-K_*β*1_ and Pb-L_*α*2_ lines were observed at the top-on position of the lead target. The high flux of the electron beam could be potentially useful for applications in material sciences.

## Figures and Tables

**Table 1:** Design parameters of plasma focus system.

Component	Length (cm)	Diameter (cm)	Material
Hollow anode	18.00	1.90/1.60 (O.D/I.D)	Copper
Cathode rod	27.20	0.95	Copper
Insulator sleeve	5.00	2.00	Pyrex
Faraday cup plate	0.1	0.75	Copper
Photodiode (BPX65)	—	—	—

**Table 2:** An array of five PIN diodes exposed with Al foil plus aluminized Mylar (*μ*m).

PIN diode	Filter	Thickness (*μ*m)
1	Aluminized Mylar	23
2	Al + aluminized Mylar	23 + 20
3	Al + aluminized Mylar	23 + 30
4	Al + aluminized Mylar	23 + 40
5	Al + aluminized Mylar	23 + 100
